# COVID-19–Related Posttraumatic Stress Disorder Among Jordanian Nurses During the Pandemic

**DOI:** 10.1017/dmp.2021.199

**Published:** 2021-06-17

**Authors:** Mohammed Qutishat, Loai Abu Sharour, Kholoud Al-Dameery, Ibtisam Al-Harthy, Sulaiman Al-Sabei

**Affiliations:** 1 Community and Mental Health Department, College of Nursing, Sultan Qaboos University (SQU), Muscat, Oman; 2 Faculty of Nursing, Al-Zaytoonah University of Jordan, Amman, Jordan; 3 Fundamentals and Administration Department, College of Nursing, Sultan Qaboos University (SQU), Muscat, Oman

**Keywords:** PTSD, nursing, coronavirus, pandemic, infectious disease

## Abstract

**Background:**

The coronavirus disease 2019 (COVID-19) outbreak has been declared a pandemic and has affected both patients and health-care workers. This study was conducted to explore the extent of posttraumatic stress disorder (PTSD) experiences among nurses because of the COVID-19 pandemic in Jordan.

**Methods:**

This study used a cross-sectional study design with a convenience sampling approach. A sample of 259 participants completed the study questionnaires, including a socio-demographic questionnaire and the Posttraumatic Stress Disorder Checklist for DSM-5 between May and July 2020.

**Results:**

The prevalence of PTSD among the study participants was 37.1%. Most study participants who exhibited PTSD symptoms presented the lowest level of PTSD (17%). The results showed significant differences in overall COVID-19-related PTSD according to the participant’s age (F = 14.750; *P* = 0.000), gender (F = 30.340; *P* = 0.000), level of education (F = 51.983; *P* = 0.000), years of experience (F = 52.33, *P* = 0.000), place of work (F = 19.593; *P* = 0.000), and working position (F = 11.597; *P* = 0.000), as determined by 1-way ANOVA.

**Conclusions:**

Nurses must be qualified and accredited to cope with reported PTSD cases and their consequences in relation to COVID-19 outbreaks. A close collaboration with a multidisciplinary team is required to recognize, manage, and encourage safety literacy among health-care professionals and individuals diagnosed with or suspected of PTSD due to COVID-19 outbreaks or any other viral outbreaks.

Coronaviruses are a family of viruses that are responsible for the common cold, in addition to more severe diseases, including acute severe respiratory syndrome (SARS) and Middle East respiratory syndrome (MERS). Scientists identified a new coronavirus as the cause of a 2019 outbreak first reported in Wuhan, China.^1^ The novel coronavirus disease 2019 (COVID-19) outbreak has been declared a public health emergency and has attracted considerable worldwide attention,^2^ infecting over 24 million people and causing around 825,000 deaths worldwide as of August 2020. Under COVID-19, many countries across the globe took stringent steps and introduced an urgent lockdown and/or curfew to deter further dissemination and bring this infectious disease under control.^3^ People who develop symptoms of COVID-19 or show similar symptoms are quarantined for 14 d.^4^ Other people have been forced to stay home with minimum physical mobility and interactions within their hometowns. Jordan announced its lockdown on March 17.

Four days of completed curfew did not stabilize the problem, and the government eventually relaxed its actions to encourage people to move from 10 am to 6 pm for shopping in local stores only, and then eventually allowed online and delivery services to do so. By reaching case 400, the lockdown included a ban on the individual who owns a car, except healthcare service, military system employees, and governmental decision-makers.^5^

In May, the Jordanian government eased the lockdown and reopened the economy, taking into consideration the short- and long-term financial and social effects of the lockdown. As a result, some industries and stores have been able to restart work steadily. However, social and cultural events, including teaching, sports, public gatherings, cultural events, tourism, and prayers in the mosque and the church, remained banned.

The numbers of infectious cases and casualties have continued to rise, which, besides a shortage of healthcare professionals and medical services across the world and a lack of consistent and accurate media reporting regarding the virus, has caused profound distress induced by confusion and uncertainty, which can impact the psychological well-being of health-care workers and cause further distress, anxiety, and mental disorders such as posttraumatic stress disorder (PTSD).^6^

Post traumatic stress disorder accompanies traumatic incidents outside the context of common human encounters, such as aggressive physical assaults, violence, illness, natural disasters, and recovery from some diseases.^7^

People with PTSD experience disturbing thoughts or emotions linked to a traumatic incident long after the event occurred. A traumatic incident can be encountered or observed personally, or may even be derived through knowledge of a traumatic event endured by a close individual.^7^ PTSD is diagnosed after a person endures symptoms for at least 1 months after a traumatic incident. Symptoms more commonly appear within 3 months after the event, but can take years to build up.^8^ A broad range of signs are associated with PTSD, such as re-experiencing painful memories, recurrent recollections, flashbacks, nightmares, mental numbness, and avoiding individuals, environments, and trauma-related incidents, as well as heightened arousal such as difficulties sleeping and processing, becoming jumpy, and feeling anxious and annoyed.^7^

Previous studies have indicated that nurses are in the frontline for developing PTSD,^9^ with ICU nurses presenting a higher prevalence of PTSD symptoms compared with other nurses.^10^ During the SARS epidemic and the H1N1 pandemic, the prevalence of PTSD among nurses ranged between 17 and 33%.^11,12^ PTSD has a dramatic effect on nurses’ perception of their work, work environment, quality of care, and health-related quality of life.^13^ During the COVID-19 pandemic, these numbers will undoubtedly increase, as there is a shortage of safety equipment for hospital staff virtually all over the world, besides daily exposure to infection, deaths, doubts about working alongside fellow staff members who might be a COVID-19 case, anxiety about separation, strict measures, and loss of medical staff, which brings more strain to this already high-pressure task and increases the probability of anxiety and fear.^14,15^

Because COVID-19 is an emerging disease, more research is required on the psychological experience of frontline nurses battling COVID-19, especially as the medical environment and community differ between countries. Previous articles have reported the prevalence of the disease and its clinical symptoms, diagnosis, and management.^16^ Many studies have also called attention to the extent of psychological conditions experienced by medical workers, such as anxiety and depression.^6^ However, limited research has focused on addressing and investigating PTSD related to the COVID-19 pandemic. The current study aimed to address this lack of knowledge by investigating the extent of nursing experiences of PTSD related to the COVID-19 pandemic in Jordan.

## Methods

Approval to conduct the study was obtained from the Research Ethics Committee of the College of Nursing at Al-Zaytoonah University, Jordan. A descriptive correlational study design was used to achieve the research objectives among Jordanian nurses. In the current study, the target population was Jordanian nurses working with patients diagnosed with COVID-19.

Convenience sampling was used to select the study population. The sample size was calculated using the rule of thumb method, found to be 180 participants.^17^ The sample comprised nurses who were willing to participate in the study, and excluded all non-Jordanian nurses, other medical health team workers, and nursing students. An online survey method was used in the current study. The researchers prepared and presented the research questions through Google Forms, which were distributed through social media platforms and could be accessed by means of a link.

All participants provided written informed consent. The nature, intent, method, and potential benefits of the study were clarified, and participants were assured that their participation in the survey was entirely voluntary and anonymous. The participants provided an in-depth self-report questionnaire on their demographic background, career history, and PTSD experiences. The researchers distributed the survey between May and June 2020, and it took approximately 10-15 min to fill out. All data were treated with confidentiality, and no access to the data was allowed except for the researchers themselves.

### Study Instruments

A self-report instrument was used as a measurement tool to investigate the extent of the research phenomena. It consisted of 2 sections: (1) demographic data, and (2) Posttraumatic Stress Disorder Checklist for DSM-5 (PCL-5).

### Posttraumatic Stress Disorder Checklist for DSM-5 (PCL-5)

The PTSD Checklist for DSM-5 (PCL-5) is a 20-item self-report measurement tool that assesses the presence and severity of PTSD symptoms. Items on the PCL-5 correspond to the DSM-5 criteria for PTSD. The PCL-5 can quantify and monitor symptoms over time, screen individuals for PTSD, and help make a provisional or temporary diagnosis of PTSD.^18^ The participants were asked to rate their responses to 20 items using a 5-point Likert scale ranging from 0 “not at all” to 4 “extremely.” The score ranged from 0 to 80, and a PCL-5 cutoff point of 33 was considered a reasonable value to use for a provisional PTSD diagnosis. PCL-5 scores exhibited strong internal consistency (α =.94), and test-retest reliability (r =. 82). The reliability of the tool was assessed on our sample and showed a high Cronbach’s α value (. 989). The PCL-5 tool is freely available online through a link.

### Statistical Analysis

SPSS Statistics (version 24.0 released in 2016; IBM SPSS Statistics for Windows; IBM Corp., Armonk, NY) was used for statistical analysis, and a *P*-value of < 0.05 was considered significant. Data are presented as mean and percentage. Analysis of variance (ANOVA) was performed to identify significant statistical differences between the study variables.

## Results

### Socio-demographic Characteristics

A sample of 259 participants completed the study survey. The age of the participants ranged from 23 to 58 y, with more than half aged between 25 and 34 y old (53.3%; *n* = 138). Around half of the participants were male (52.1%; *n* = 135), and most them were married (80.3%; *n* = 208). Table 1 details these results.

**Table 1.** Socio-demographic characteristics of the participants (*N* = 259)

Variable	Frequency (N)	Percentage (%)
**Age (y)**		
Less than 25	22	8.5
25-34	138	53.3
35-44	44	17.0
45-54	33	12.7
Above 54	22	8.5
**Gender**		
Male	135	52.1
Female	124	47.9
**Marital status**		
Single	51	19.7
Married	208	80.3
**Level of education**		
Diploma	73	28.2
Bachelor	161	62.2
Postgraduate	25	9.7
**Years of experience**		
Less than 3	17	6.6
3-6	90	34.7
7-10 y	88	34.0
More than 10	64	24.7
**Working place**		
Government	77	29.7
Private	162	62.5
Military	20	7.7
**Position**		
RN	109	42.1
In-charge	73	28.2
Head nurse	26	10.0
Education	51	19.7
**Department**		
Emergency	85	32.8
Medical units	70	27.0
Surgical units	39	15.1
Critical units	48	18.5
Mental health units	17	6.6

### Prevalence and Level of PTSD Related to COVID-19 Case Exposure

Regarding COVID-19 case exposure, most participants showed their workplace had received a COVID-19 case (74.5%; *n* = 193), with 53.3% (*n* = 138) reporting that they had been in direct contact with the patient. The results also showed that 56.4% of participants (*n* = 146) had a family member or friend who was infected or suspected of having a COVID-19 infection, and 11.2% (*n* = 29) of participants had been quarantined (Table 2).

**Table 2.** Prevalence and levels of COVID-19–related PTSD case exposure (*N* = 259)

Variable	Frequency (*N*)	Percentage (%)
**Are any of your family members or friends suspected of having COVID-19 infection?**		
Yes	91	35.1
No	146	56.4
Do not know	22	8.5
**Did your place of work receive any COVID-19 cases?**		
Yes	193	74.5
No	66	25.5
**Were you in direct contact with any patient who was confirmed to have COVID-19?**		
Yes	138	53.3
No	121	46.7
**Did you force any type of quarantine?**		
Yes	29	11.2
No	230	88.8

A composite score for each tool was calculated by summarizing the nurses’ responses to the questionnaire. The prevalence of PTSD among the study participants was estimated, with 37.1% (*n* = 96) of participants reporting a score indicative of PTSD. The level of PTSD among the study participants was divided into 3 categories: high level of PTSD, medium level of PTSD, or low level of PTSD. This criterion was adopted where the PTSD score exceeded the cutoff point of 33, calculated according to the formula (highest grade - lowest grade) divided by 3. Thus, the level of PTSD was split into 3 levels: low level from 33 to 48.6 points, medium level from 48.6 to 64.2 points, and high level from 64.2 to 80 points. The result of this study show that most of the study participants who exhibit PTSD symptoms were at the lowest level of PTSD (17%; *n* = 44) compared with the high (10.8%; *n* = 28) and moderate (8.5%; *n* = 22) levels (Table 3).

**Table 3.** Level of PTSD among the study participants due to COVID-19 pandemic (*N* = 259)

Level of PTSD	Frequency (*N*)	Percent (%)
Not a PTSD	165	63.7
Low level of PTSD	44	17.0
Moderate level of PTSD	22	8.5
High level of PTSD	28	10.8
Total	259	100.0

### Differences in COVID-19–Related PTSD According to Socio-demographic Characteristics

The results of the current study indicate that there were significant differences in overall COVID-19-related PTSD, according to the participant’s age (F = 14.750; *P* = 0.000), gender (F = 30.340; *P* = 0.000), and level of education (F = 51.983; *P* = 0.000). Differences in COVID-19-related PTSD subdomains according to the participants’ socio-demographic characteristics were evaluated in the current study, and the results are presented in Table 4.

**Table 4.** Distribution of PTSD experiences based on the participants’ demographic characteristics (*N* = 259)

Variable	Re-experiencing Mean (SD) *P*-value	Avoidance Mean (SD) *P*-value	Arousal Mean (SD) *P*-value	Negative thoughts and beliefs Mean (SD) *P*-value	Overall significant
**Age (y)**					
Less than 25	15.00 (0.00)	6.00 (0.00)	17.00 (0.00)	15.00 (0.00)	70.00 (0.00)
25-34	6.41(7.812)	2.42 (3.02)	7.41(10.75)	6.09 (9.39)	40.13 (28.83)
35-44	8.50 (2.53)	3.00 (1.01)	10.00(2.02)	7.50 (0.51)	47.00 (4.05)
45-54	6.51(1.33)	3.88 (0.33)	1.36 (0.99)	3.88 (0.33)	34.51 (0.00)
Above 54	11.00 (0.00)	5.00 (0.00)	18.00 (0.00)	11.00 (0.00)	61.00 (0.00)
	F = 12.453 *P* = 0.00*	F = 16.835 *P* = 0.00*	F = 21.739 *P* = 0.00*	F = 11.520 *P* = 0.00*	F = 14.750 *P* = 0.00*
**Gender**					
Male	9.90 (6.73)	3.38 (2.87)	12.09 (10.16)	9.41(8.34)	52.04 (25.89)
Female	5.71(5.10)	3.06 (2.08)	5.20 (6.140)	4.83 (5.40)	37.11 (17.44)
	*F* = 30.340 *P* =0.00*	F = 0.996 *P* = 0.319	F = 42.743 *P* = 0.00*	F = 26.911 *P* = 0.00*	F = 30.340 *P* = 0.00*
**Marital status**					
Single	6.47(7.50)	2.58 (3.00)	7.90 (8.00)	8.74 (5.50)	43.84 (23)
Married	8.25 (6.00)	3.38 (2.37)	9.01(9.40)	6.85 (7.81)	45.15 (23.52)
	F = 3.246 *P* = 0.073	F = 4.127 *P* = 0.043**	F = 0.606 *P* = 0.437	F = 0.606 *P* = 0.437	F = 0.217 *P* = 0.642
**Level of education**					
Diploma	10.91(5.97)	3.98 (2.62)	12.49 (9.63)	10.04 (7.94)	54.68 (23.97)
Bachelor	2.41(2.52)	1.80 (1.67)	3.31(3.34)	2.11(3.23)	28.04 (10.20)
Postgraduate	4.48 (3.44)	2.56 (1.96)	1.00 (0.00)	4.00 (0.00)	31.04 (5.39)
	F = 78.725 *P* =0.00*	F = 22.766 *P* = 0.00*	F = 48.382 *P* = 0.00*	F = 40.767 *P* = *0*.00*	F = 51.983 *P* = 0.00*

*P < 0.01.

**P < 0.05.

### Differences in COVID-19–Related PTSD According to Work-related Factors

Regarding work-related factors, a significant difference was reported in COVID-19–related PTSD according to the participants’ years of experience, place of work, and working position (F = 52.333; *P* = 0.000; F = 19.593; *P* = 0.000; and F = 11.597; *P* = 0.000, respectively). Detailed results are presented in Table 5.

**Table 5.** Distribution of PTSD experiences based on the participants’ career history (*N* = 259)

Variable	Re-experiencing Mean (SD) *P*-value	Avoidance Mean (SD) *P*-value	Arousal Mean (SD) *P*-value	Negative thoughts and beliefs Mean (SD) P-value	Overall significant
**Years of experience**					
less than 3	15.00 (0.00)	6.00 (0.00)	17.00 (0.00)	21.00 (0.00)	70.00 (0.00)
3-6	8.24(4.92)	3.28 (2.023)	8.52 (7.83)	12.59 (5.98)	44.42 (18.57)
7-10	5.84 (8.75)	2.57(3.43)	8.48 (12.06)	13.63 (10.21)	42.25 (32.56)
More than 10	8.35 (2.26)	3.33 (1.155)	7.42 (5.86)	11.48 (3.44)	42.52 (10.13)
	F = 11.660 *P* = 0.00*	F = 9.794 *P* = 0.00*	F = 5.412 *P* = 0.00*	F = 8.319 *P* = 0.00*	F = 52.333 *P* = 0.00*
**Working place**					
Government	4.67 (3.79)	1.92 (1.62)	4.09 (2.80)	3.87 (2.34)	32.91(7.63)
Private	9.55 (6.80)	3.92 (2.65)	11.35 (10.23)	9.12 (8.46)	51.39 (26.20)
Military	6.90 (5.60)	2.60 (2.23)	6.20 (7.94)	4.70 (6.28)	38.40 (19.75)
	F = 17.726 *P* = 0.00*	F = 19.586 *P* = 0.00*	F = 19.728 *P* = 0.00*	F = 15.946 *P* = 0.00*	F = 19.593 *P* = 0.00*
**Position**					
RN	10.74 (7.65)	3.87 (3.23)	11.94 (11.39)	9.68 (10.31)	53.50 (30.65)
In-charge	3.92 (4.76)	2.11(2.08)	6.72 (7.50)	4.90 (4.36)	35.45 (16.90)
Head- nurse	9.77 (2.94)	2.15 (0.37)	10.76 (2.94)	7.23 (1.84)	47.92 (7.35)
Education	6.57 (.50)	4.00 (0.00)	4.01(3.50)	5.29 (1.50)	38.45 (4.00)
	F = 23.075 *P* = 0.00*	F = 11.566 *P* = 0.00*	F = 11.119 *P* = 0.00*	F = 8.081 *P* = 0.00*	F = 11.597 *P* = 0.00*
**Department**					
Emergency	7.94(6.12)	3.25 (2.43)	8.95 (8.77	7.22 (7.11)	45.11 (22.43)
Medical units	7.76 (6.34)	3.17(2.53)	8.36 (9.14)	7.07(7.42)	44.20 (23.33)
Surgical units	8.36 (6.68)	3.30 (2.73)	9.48 (9.73)	7.79 (7.85)	46.53 (24.75)
Critical units	7.58 (6.98)	3.22 (2.74)	8.50 (10.07)	7.15 (8.46)	44.18 (26.16)
Mental health units	8.12 (5.41)	3.11(2.14)	9.05(7.55)	6.76(5.59)	44.82 (18.81)
	F = .094 *P* = 0.984	F = 0.029 *P* = 0.998	F = 0.089 *P* = 0.986	F = 0.081 *P* = 0.988	F = 0.075 *P* = .0990

*P < 0.01.

### Distribution of PTSD Experiences Based on Case Exposure

Regarding the case exposure of nurses, a significant difference was reported in COVID-19–related PTSD according to the participants’ close contact with a relative/friend with COVID-19 or infection/suspicion of COVID-19 (F = 10.522; *P* = 0.000), if their workplace had received COVID-19 cases (F = 31.059; *P* = 0.000), and if they were forced to quarantine (F = 361.30; *P* = 0.000). Detailed results are presented in Table 6.

**Table 6.** Distribution of PTSD experiences based on the participants’ COVID-19–related PTSD case exposure

	Re-experiencing Mean (SD) P-value	Avoidance Mean (SD) P-value	Arousal Mean (SD) P-value	Negative thoughts and beliefs Mean (SD) P-value	Overall significant
**Are any of your family members or friends suspected of having COVID-19 infection?**					
Yes	11.60 (5.92)	4.59 (2.48)	10.6 (11.97)	9.37(10.13)	53.62 (28.46)
No	5.87 (6.06)	2.26 (2.31)	7.73 (7.50)	5.91 (5.49)	39.73 (19.88)
Don’t Know	6.00 (0.00)	4.00 (0.00)	8.00 (0.00)	7.00 (0.00)	43.00 (0.00)
	F = 29.098 *P* = 0.00*	F = 30.886 *P* = *0*.00*	F = 3.079 *P* = 0.048	F = 6.309 *P* = 0.002*	F = 10.522 *P* = *0*.00*
**Did your place of work receive any COVID-19 cases?**					
Yes	6.95 (6.78)	2.62 (2.62)	6.90 (9.54)	5.93 (8.01)	40.41 (24.74)
No	10.66 (3.71)	5.00 (0.822)	14.33 (4.53)	11.00 (3.29)	58.00 (11.31)
	F = 17.932 *P* = 0.00*	F = 52.359 *P* = 0.00*	F = 35.410 *P* = 0.00*	F = 24.935 *P* = 0.00*	F = 31.059 *P* = 0.00*
**Were you in direct contact with any patient who was confirmed to have COVID-19?**					
Yes	9.31(6.64)	9.32 (6.64)	9.05 (10.50)	8.21(8.44)	47.64 (25.84)
No	6.28 (5.59)	6.28 (5.59)	8.49 (7.32)	6.10 (5.93)	41.76 (19.87)
	F = 15.599 *P* = 0.00*	F = 0.051 *P* = 0.822	F = 0.243 *P* = 0.622	F = 5.277 *P* = 0.02	F = 3.598 *P* = 0.059
**Did you force any type of quarantine?**					
Yes	20.0 (0.00)	8.00 (0.00)	28.0 (0.00)	24.0 (0.00)	95.00 (0.00)
No	6.37 (4.95)	2.62 (1.98)	6.37 (6.44)	5.11 (4.71)	38.57 (16.07)
	F = 218.82 *P* = 0.00*	F = 212.40 *P* = 0.00*	F = 325.76 *P* = 0.00*	F = 464.20 *P* = 0.00*	F = 361.3 *P* = 0.00*

*P < 0.01.

## Discussion

There have been few studies on the experience of PTSD symptoms in nurses due to infectious diseases such as COVID-19, particularly among Jordanian nurses. This study represents a step toward estimating the prevalence of PTSD among Jordanian nurses as a result of the COVID-19 pandemic and the governmental restrictions and quarantine in Jordan. This study aimed to explore the extent of nursing experiences of PTSD in relation to the COVID-19 pandemic in Jordan. The prevalence of PTSD among the study participants was 37.1%. The majority of them were at the lowest level of PTSD (17%), which is 3 times higher than the prevalence of PTSD among medical health workers in Singapore in 2020^19^ and 3 times higher than the prevalence of PTSD among health team workers during the SARS pandemic.^11^ Although the COVID-19 pandemic has some parallels to the SARS virus, it has become more severe and has contributed to more hospitalizations and deaths worldwide. In addition, COVID-19 tends to grow exponentially across the world, impacting more people including health team workers every day in several ways (eg, financial losses, work overload, difficulties getting critical supplies, rising social alienation, confusion, etc.).^20^ This could be attributed to decreased mental preparedness and fewer qualified health nurses to deal with emergencies such as the rapid spread of infectious disease, together with the exaggerated role of media and social interaction in discussing disease facts and accurate case numbers.^21,22^ As a result, the mental health effects of the COVID-19 pandemic will be more severe and more serious than those of the SARS outbreak.

Another consideration is related to an increased sense of personal risk as COVID-19 is correlated with high morbidity and can be lethal in some patients. Anticipated stock shortages and an increase in suspected and confirmed reports of COVID-19 further contribute to stress and fear among health professionals.^23^ Another factor is related to the safety measures taken by the Jordanian government; as soon as reports of a novel coronavirus in China appeared in early 2020, the National Epidemics Committee and the Ministry of Health in Jordan approved several protocols in January to cope with the arrival of coronavirus to the world. Just a few days after learning that coronavirus-infected patients had taken part in a wedding in the city of Irbid, north of the capital Amman, on March 17, the government was forced to announce a quarantine and lockdown. These precautions were later described as one of the strictest measures in the world, impacting every aspect of individuals’ lives, including their physical and emotional status.^5^

Nurses need to understand that this health crisis may persist for some time. Therefore, there is a need for training and qualifying the nurses to manage suspected cases of PTSD associated with the COVID-19 outbreak and its consequences. The goal would be to help them collaborate effectively with a multidisciplinary team to assess, treat, educate, and promote health awareness among individuals diagnosed with PTSD or suspected of having PTSD due to the COVID-19 outbreak and any further infectious outbreaks and their protocols and measures.^24^ The results of the current study highlight significant differences in overall COVID-19–related PTSD according to participants’ age, gender, and level of education, which is similar to previous studies that assessed PTSD among nursing staff in China related to the COVID-19 pandemic.^25^ Unlike earlier studies,^1^ our investigation highlighted that being a male nurse was associated with a higher PTSD level associated with the COVID-19 pandemic. This observation may be explained by the tendency of females to seek more social support than males; in stressful situations, females may use a tend-and-befriend response, a behavior exhibited mainly by females in response to a threat.^26^ It refers to the protection of offspring (tending) and seeking out their social group or mutual defense (befriending).^26^

Our investigation indicated that younger nurses with lower educational qualifications and clinical experience had higher levels of PTSD compared with others, which is in line with previous studies,^25,27^ While younger nurses with inadequate credentials are more likely to display a lower degree of competence, making them more prone to anxiety and mental disengagement than other health team members, frontline nurses who handle their dedication have physically and mentally challenged patients with infectious diseases, delivering high-quality clinical services for patients.^28^ Moreover, at the early stage of the infectious epidemic, junior nurses may not have received adequate warning about exposure or may not have been provided with adequate protection, leading them to potentially put themselves at higher risk, either physically or psychologically. For younger nurses with fewer qualifications and less experience, when exposed to any new infectious disease, they may spend hours monitoring television and social media platforms displaying information on newly identified cases, treatment modalities, recovered instances, and even deaths, which can significantly worsen their PTSD and anxiety.^21,22^ Thus, it is highly recommended that junior nurses are offered reliable sources of information to avoid myths, rumors, and misinformation that can feed into their anxiety. These reliable resources include the World Health Organization (WHO), the Centre’s for Disease Control and Prevention (CDC), the Centre for Health Security, and the National Health Service, among others.

Regarding work-related factors, a significant difference was reported in COVID-19–related PTSD according to participants’ workplace and working position, which is consistent with the findings of other studies.^27^ Our results indicated that working in the private sector or as a registered nurse are associated with high levels of PTSD because of the COVID-19 outbreak. This finding can be explained by the need for these nurses to have a comprehensive knowledge, skill-set, and attitude that promotes patients’ well-being and enhances their care outcome. During the COVID-19 pandemic and the process of quarantine and lockdown, the Jordanian government and the Jordanian Ministry of Health allowed few private hospitals to test or treat COVID-19 cases, limiting case exposure to those nurses in the public and military hospitals^29^ while putting private nurses under extensive pressure related to dealing with undiscovered cases and limiting their clinical experience of COVID-19 exposure and treatment. It is vitally important to provide high-quality psycho-education and skills training for registered nurses on how to manage virus-related uncertainties and on updates to rapidly changing treatment protocols, which may be challenging for some nurses who are exposed to fewer COVID-19 cases.^30^

Regarding the nurses’ case exposure, our results indicate a significant difference in COVID-19–related PTSD according to the participants’ close contact with a relative/friend with COVID-19 or infection/suspicion of COVID-19, receiving COVID-19 cases at their workplace, and being forced to quarantine, highlighting that nurses who have a suspected family member or friend with COVID-19 (mean score of 53.62) or who were forced to quarantine (mean score of 95.00) have higher levels of PTSD, consistent with previous studies.^3,31^ This may due to the reason that nurses at their homes may develop fear of transmitting the virus to their family due to COVID-19 and had trouble and difficulties in isolation-related life.^32^ This kind of fear and anxiety may lead to high level of uncertainty and challenging leading people to experience a higher adaptation difficulty,^33^ which was also detected during the SARS outbreak.^34^

Nurses serving at the frontline during the pandemic are critical, rendering them more vulnerable to distress and fatigue related to daunting health-care services, in addition to fear of infection,^35^ being hospitalised,^36^ or transmitting the infection from or to their family members.^37^ According to a new study, nurses’ anxiety is worsened by exposure to life-threating viral infections, the risk of infecting their family, and concerns regarding family care and responsibilities.^37^

Combining a stress-reduction intervention with an educational program for enhancing hardiness and the adoption of protective mental health measures before working under such stressful conditions might be more effective in improving the mental health status of nurses during an infectious epidemic.^31,38^

The results of this study make a significant contribution to the existing literature on COVID-19 by presenting the experience of Jordanian nurses during the COVID-19 pandemic, specifically related to PTSD symptoms. However, several limitations need to be considered. The study was performed early in the COVID-19 outbreak and it was only performed in Jordan through online surveys, which may limit the generalizability of the findings. The study also failed to assess the mental health history of the nurses surveyed, which may affect the results of their participation. Perhaps the limited interaction with COVID-19 patients in the hospital at the time of data collection resulted in the inability to observe the correlation between clinical psychiatric morbidity and contact with COVID-19 patients. Previous studies have shown that posttraumatic morbidity represented a dynamic psychosocial reaction, not just immediate exposure, but also other contextual influences.^39,40^ On the other hand, coping mechanisms have been shown to moderate the psychological effects of extremely traumatic situations.^41^ Psychiatric comorbidity workers have been most often faced with strategies focused on negative emotions, such as anxiety, denial, and confusion, which are often not discussed in our inquiries.

Follow-up studies could assess the progression or even a potential rebound effect of psychological manifestations once the imminent threat of COVID-19 subsides. Future follow-up studies may better determine the persistence or even the possible recovery of psychological conditions as the immediate danger of COVID-19 subsides. Future studies should analyze more predictors for PTSD related to an infectious disease outbreak, such as the presence of comorbid conditions, the impact of self-expectation, self-esteem, social support, coping measures, and the efficiency of the health-care system. Future studies should also consider the mental health history of the participants. This could be gathered by means of the socio-demographic questionnaire in follow-up surveys.

## Conclusions

This study aimed to explore the extent of nursing experiences of PTSD during the COVID-19 pandemic in Jordan. Our study revealed that significant PTSD affects a sizeable minority of Jordanian nurses facing the consequences of the COVID-19 pandemic. Some variables associated with the severity of PTSD were male gender, younger age, lower educational qualifications and clinical experience, working in the private sector, or working as a registered nurse, which were all associated with a higher level of PTSD. Nurses need to be trained and certified to handle suspected cases of PTSD associated with COVID-19 outbreaks and their effects. They also need to work efficiently with a multidisciplinary team to identify, treat, and promote health awareness among health-care providers and individuals diagnosed with PTSD or suspected of PTSD as a result of COVID-19 outbreaks, as well as any future infectious outbreaks.
